# MATE-Type Proteins Are Responsible for Isoflavone Transportation and Accumulation in Soybean Seeds

**DOI:** 10.3390/ijms222112017

**Published:** 2021-11-06

**Authors:** Ming-Sin Ng, Yee-Shan Ku, Wai-Shing Yung, Sau-Shan Cheng, Chun-Kuen Man, Liu Yang, Shikui Song, Gyuhwa Chung, Hon-Ming Lam

**Affiliations:** 1 Centre for Soybean Research of the State Key Laboratory of Agrobiotechnology and School of Life Sciences, The Chinese University of Hong Kong, Hong Kong, China; sammingsin0212@gmail.com (M.-S.N.); vincentyungws@hotmail.com (W.-S.Y.); chengsaushan@yahoo.com (S.-S.C.); chunkuenman@cuhk.edu.hk (C.-K.M.); yangl600@163.com (L.Y.); 2 Institute of Advanced Agricultural Sciences, Peking University, Beijing 100871, China; shikui_song@163.com; 3 Department of Biotechnology, Chonnam National University, Yeosu 59626, Korea; Chung@chonnam.ac.kr

**Keywords:** soybean, seed, isoflavone, multidrug and toxic compound extrusion (MATE) transporter, genistein, daidzein, glycitein

## Abstract

Soybeans are nutritionally important as human food and animal feed. Apart from the macronutrients such as proteins and oils, soybeans are also high in health-beneficial secondary metabolites and are uniquely enriched in isoflavones among food crops. Isoflavone biosynthesis has been relatively well characterized, but the mechanism of their transportation in soybean cells is largely unknown. Using the yeast model, we showed that GmMATE1 and GmMATE2 promoted the accumulation of isoflavones, mainly in the aglycone forms. Using the tobacco BrightYellow-2 (BY-2) cell model, GmMATE1 and GmMATE2 were found to be localized in the vacuolar membrane. Such subcellular localization supports the notion that GmMATE1 and GmMATE2 function by compartmentalizing isoflavones in the vacuole. Expression analyses showed that GmMATE1 was mainly expressed in the developing soybean pod. Soybean mutants defective in GmMATE1 had significantly reduced total seed isoflavone contents, whereas the overexpression of GmMATE1 in transgenic soybean promoted the accumulation of seed isoflavones. Our results showed that GmMATE1, and possibly also GmMATE2, are *bona fide* isoflavone transporters that promote the accumulation of isoflavones in soybean seeds.

## 1. Introduction

While rich in proteins and oils, soybean seeds are also uniquely rich in isoflavones, a group of naturally occurring phytoestrogens of high nutritional value. Evidence has shown that in humans, isoflavones could reduce the risks of several hormone-related cancers [1], alleviate post-menopausal symptoms [2], prevent osteoporosis [3,4], and serve as nutraceuticals [5]. In legumes, isoflavones act as chemoattractants to Rhizobia for the induction of symbiosis [6] and work as phytoalexins against pests and pathogens [7].

Genistein, daidzein, and glycitein are the unmodified isoflavone aglycones. Inside the cells, aglycones are usually converted to the more stable glycosides (genistin, daidzin, and glycitin) and malonylglycosides (malonylgenistin, malonyldaidzin, and malonylglycitin) for storage (reviewed in [8]); therefore, these isoflavones are the most commonly found in soybean seeds [9]. Isoflavones are synthesized by isoflavone synthases (IFS) 1 and 2 [10] using the substrates generated by the phenylpropanoid pathway. IFS catalyses the oxidation of 4′,7-dihydroxyflavanone (liquiritigenin) or 5,7,4′-trihydroxyflavanone (naringenin) to form intermediates which are ultimately converted to daidzein and genistein, respectively. Glycitein and daidzein are both derived from liquiritigenin. Besides IFS, the conversion of liquiritigenin to glycitein involves other enzymes, including flavanone-6-hydroxylase and isoflavone methyltransferase [11]. While the biosynthetic pathway of isoflavones in soybeans has been well dissected [12], the identities of isoflavone transporters in soybeans remain unclear.

Multidrug and toxic compound extrusion (MATE) is one of the five major transporter superfamilies in living organisms. In plants, MATE transporters comprise of 117 gene family members in *Glycine max* [13], 56 in *Arabidopsis thaliana* [14], 49 in *Zea mays* [15], 71 in *Populus trichocarpa* [16], 67 in *Solanum lycopersicum* [17], 53 in *Oryza sativa* [18], 65 in *Vitis vinifera* [19], 33 in *Vaccinium corymbosum* [20], 48 in *Solanum tuberosum* [21], and over 40 in *Medicago truncatula* [22]. MATEs are characterized by 12 transmembrane α-helical domains, which are distinct from other solute transporters. MATE transporters are substrate/proton antiporters or substrate/Na^+^ antiporters [23] which function in toxin removal or the accumulation of secondary metabolites through secondary active transport [24]. Emerging studies have shown that plant MATE transporters can translocate a broad range of substrates such as heavy metals, cations, organic acids, secondary metabolites, and hormones across the membranes of different organelles including Golgi apparatus, endoplasmic reticulum (ER), and vacuolar membrane against the electrochemical gradient [25,26,27]. 

MATE transporters are also involved in the transportation of flavonoids. For example, the *A. thaliana* AtTT12 acts as a flavonoid/H^+^ antiporter for proanthocyanidin accumulation in the seed coat [28]. In *M. truncatula*, MtMATE1 transports epicatechin 3′-O-glucoside, which is the precursor of proanthocyanidin, while MtMATE2 was shown to transport anthocyanins and flavone glycosides [22,29]. In *Vitis vinifera*, anthoMATE1 and anthoMATE3 are localized in the vacuolar membrane for the transportation of acetylated anthocyanins into the vacuole for storage [19]. These studies inferred the important roles of MATE transporters in flavonoid accumulation.

In a previous study, we identified GmMATE1 (Glyma.19G120200) and GmMATE2 (Glyma.19G120300), which were located within the quantitative trait locus (QTL) corresponding to seed antioxidant content, phenolic content, and flavonoid content. These two MATE proteins are phylogenetically related to the flavonoid transporters in other species [30]. In this study, we characterized the isoflavone transporting ability of these two MATE proteins and showed that they were localized in the vacuolar membrane and were differentially expressed in seed pods. We also generated transgenic soybeans which overexpressed GmMATE1 and GmMATE1-defective soybean mutants to confirm the significance of the GmMATE1 transporter in regulating isoflavone accumulation in soybean seeds.

## 2. Results 

### 2.1. GmMATE1 and GmMATE2 Are Isoflavone Transporters in Soybeans

To test if GmMATE1 and GmMATE2 could transport isoflavones, a yeast uptake assay followed by quantification with ultra-performance liquid chromatography (UPLC) was performed. The coding sequences (CDSs) of GmMATE1 and GmMATE2 from the soybean accessions W05 and C08 were expressed in yeast, driven by the ADH1 promoter [31]. GmMATE1*^W05^* and GmMATE1*^C08^* differ in two non-synonymous single-nucleotide polymorphisms (SNPs) while GmMATE2*^W05^* and GmMATE2*^C08^* differ in one non-synonymous SNP [30]. The transgenic yeasts were fed different mixtures of isoflavones or flavones/flavonols/flavonoids (Figure S1) [32]. The accumulation of various secondary metabolites in the yeast was quantified by UPLC against known standards (Figure S2).

**Figure 1:**
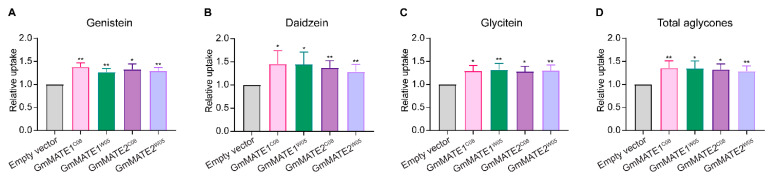
Yeast uptake assay using a mixture of genistein, daidzein, and glycitein. The level of (**A**) genistein, (**B**) daidzein, (**C**) glycitein, or (**D**) total aglycone (genistein, daidzein and glycitein) was determined. Metabolites were extracted from the yeast after feeding for 24 h and analyzed using UPLC against the respective standards. Values obtained from the yeast expressing GmMATE1 or GmMATE2 were normalized to those from the empty vector control (EV) in the same experiment. Values shown were the mean of six independent experiments ± SEM. Significant differences compared to EV were determined by Mann-Whitney test. *, *p* < 0.05; **, *p* < 0.01.

When fed with a mixture of genistein, daidzein, and glycitein, the aglycone form of these isoflavones, the transgenic yeasts expressing GmMATE1 and GmMATE2 significantly accumulated more of all three metabolites compared to the yeast transformed with the empty vector (EV) (Figure 1 and Figure S3). The allelic forms from either the cultivated soybean C08 or the wild soybean W05 exhibited similar activities.

**Figure 2:**
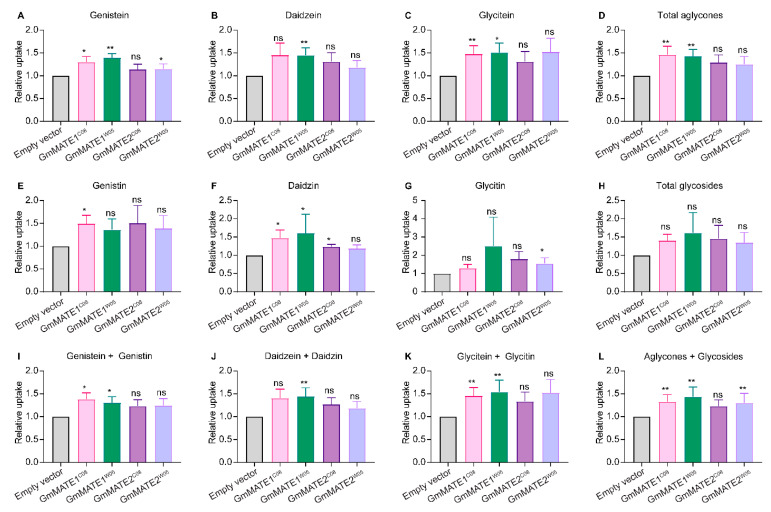
Yeast uptake assay with a mixture of genistin, daidzin, and glycitin. Metabolites were extracted from the yeast after feeding for 24 h and analyzed using UPLC against the respective standards. It appeared some of the glycosides were converted to aglycones in the yeast by endogenous enzymes. Values obtained from the yeast expressing GmMATEs were normalized to those from the empty vector control (EV) in the same experiment. The relative uptake levels of individual isoflavone species (**A**–**C,E**–**G**) and different isoflavone combinations (**D,H,I**–**L**) were determined. Total aglycones (**D**) refers to the combination of genistein, daidzein, and glyciteins. Total glycosides (**H**) refers to the combination of genistin, daidzin, and glycitin. The relative uptake level of total aglycones and glycosides is shown in (**L**). Values shown are the mean of six independent experiments ± SEM. Significant differences compared to EV were determined by Mann-Whitney test. *, *p* < 0.05; **, *p* < 0.01; ns, not significant.

When the yeast constructs were fed with a mixture of isoflavone glycosides (genistin, daidzin, and glycitin), yeasts expressing GmMATE1*^C08^* or GmMATE1*^W05^* were able to accumulate more aglycones as well as more daidzin compared to the EV-transformed yeasts. Both aglycone and glycoside forms were detected in the metabolite extracts, probably due to the activities of endogenous hydrolytic enzymes which cleaved the sugar from the glycosides in the yeast cells (Figure 2 and Figure S4). In the same experiment, although the yeasts expressing GmMATE2 also showed a similar trend in secondary metabolite accumulation to GmMATE1-expressing yeasts, the improved accumulation was not statistically significant, suggesting that GmMATE1 may have a higher activity than GmMATE2. In the experiment where the yeasts were fed with malonylglycosides, the malonylglycosides were not detected in the yeast cell extracts with or without GmMATE expressions (Figure S5). 

To further assess the specificity of the MATE proteins, the transgenic yeasts were fed with a mixture of seven flavonoid-related substrates (Figure S1) including flavonoids (fisetin, liquiritigenin, and naringenin), flavones/flavonols (quercetin, kaempferol, and myricetin), and isoflavone (formononetin) (Figure S6). There were no significant improvements in the accumulation of all seven metabolites in the transgenic yeast expressing GmMATE1 or GmMATE2 for both C08 and W05 allelic forms.

### 2.2. Both GmMATE1 and GmMATE2 Were Localized in the Vacuolar Membrane

**Figure 3:**
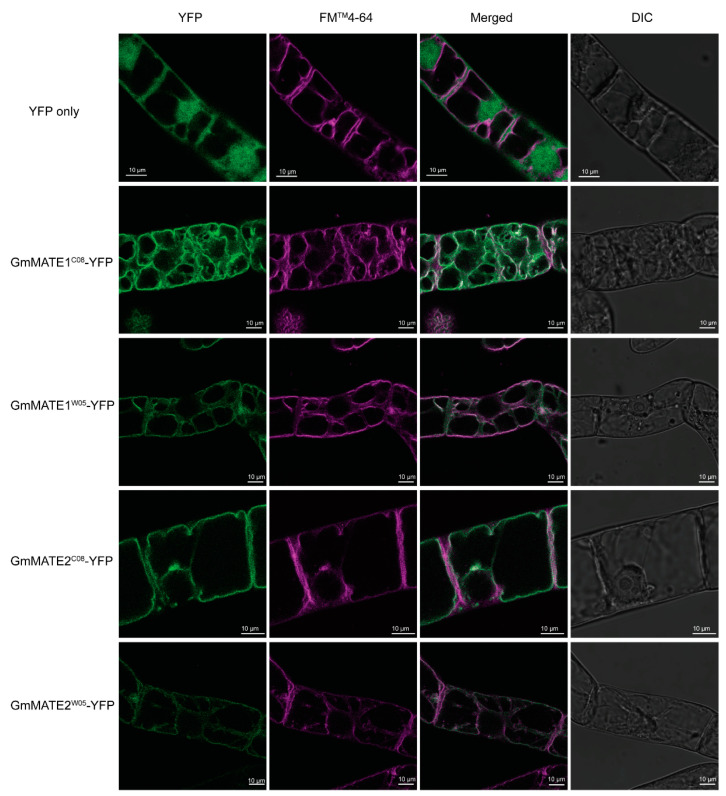
Subcellular localizations of GmMATE1-YFP and GmMATE2-YFP were determined using confocal microscopy. The cells were incubated with FM^TM^4-64 in the dark at room temperature for 24 h before imaging. DIC, differential interference contrast. Signals from GmMATE-YFP and FM^TM^4-64 were illustrated in green and magenta, respectively.

To better understand the physiological role of GmMATE1 and GmMATE2 as transporters at the cellular level, a subcellular localization study was conducted using transgenic *Nicotiana tabacum* (tobacco) BrightYellow-2 (BY-2) cells which ectopically expressed GmMATE1-YFP or GmMATE2-YFP. FM^TM^4-64 was used to visualize the plasma membrane and vacuolar membrane [33]. Upon the addition of FM^TM^4-64, when the stain first appeared on the plasma membrane, the FM^TM^4-64 signal did not overlap with the YFP signals from any of the GmMATE-YFP constructs (Figure S7), suggesting that the fusion GmMATE-YFP proteins were not localized on the plasma membrane. After 24 h of incubation, FM^TM^4-64 signals appeared in the vacuolar membrane and co-localized with the signals from GmMATE1-YFP and GmMATE2-YFP for both the C08 and W05 allelic forms (Figure 3).

Based on the results of yeast uptake assays and subcellular localization studies, the allelic differences between W05 and C08 did not seem to result in the differential accumulation of isoflavones between GmMATE^W05^ and GmMATE^C08^ expressing yeasts. Therefore, only the C08 allelic form was employed in subsequent functional analyses.

### 2.3. GmMATE1 and GmMATE2 Were Expressed in Developing Pods, Seeds, and the Seed Coat

**Figure 4:**
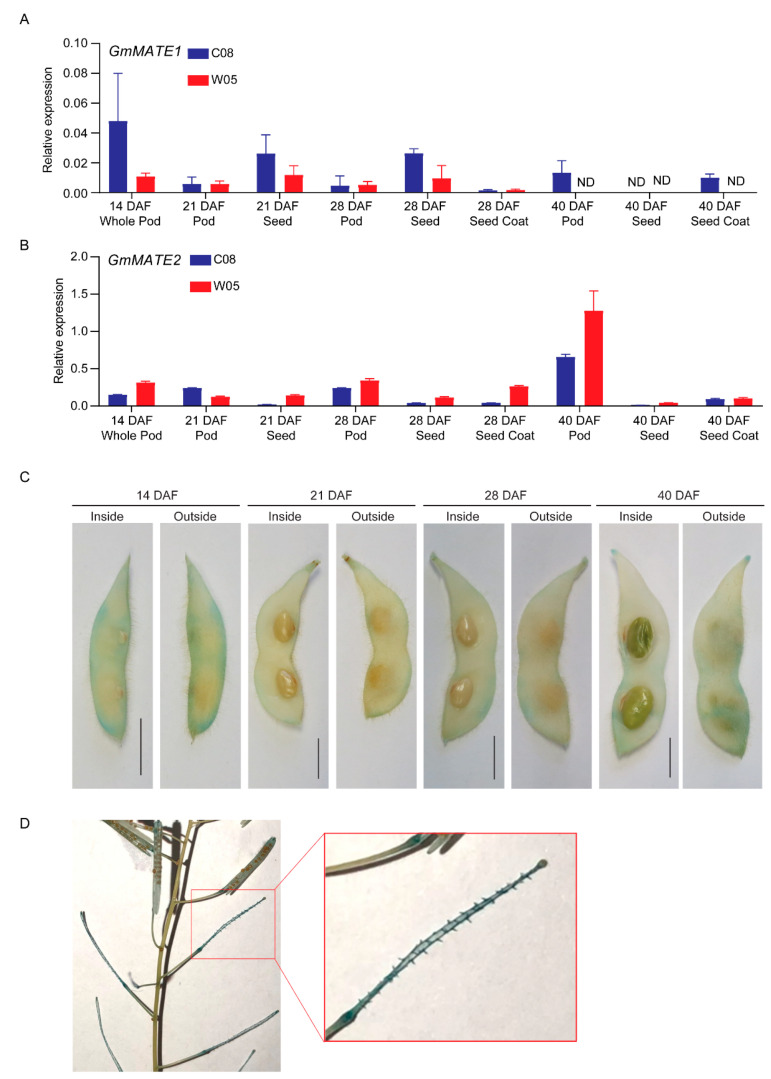
Relative expressions of GmMATE1 and GmMATE2 in different tissues within the developing soybean seed pod. The relative expression of (**A**) GmMATE1 and (**B**) GmMATE2 in different tissues of the developing pod, normalized to the reference gene, *VPS*, using the 2^−ΔCt^ method. DAF, days after flowering. Values shown are the averages of three technical replicates ± SEM. Similar expression trends were obtained in another biological repeat. (**C**,**D**) Photos showing the GUS staining of (**C**) transgenic soybean pods and (**D**) transgenic *A. thaliana* expressing *β-glucuronidase* (GUS) driven by the native GmMATE1*^C08^* promoter. Similar results were obtained from two independent transgenic lines. Scale bar = 1 cm.

To obtain more insight on the physiological functions of GmMATE1 and GmMATE2, the expression of these two genes in pods, seeds, and the seed coat at different developmental stages were quantified using RT-qPCR. Although GmMATE1 and GmMATE2 showed different expression patterns among pod, seed, and seed coat during the development; in general, the expression of GmMATE1 and GmMATE2 could be found in all three types of tissues and the expression tended to diminish 40 days after flowering (Figure 4). 

Next, we attempted to construct transgenic and mutant soybeans for GmMATE1 and GmMATE2 for tissue-specific gene expression studies, as well as gain-of function and loss-of function analyses. Only the constructs of GmMATE1 were successful. Since GmMATE1 and GmMATE2 seemed to share common functions (see above), we focused on the functional analyses of GmMATE1 subsequently.

Since RT-qPCR results above showed that GmMATE1 was expressed in developing pods, seeds, and the seed coat, we visualized its tissue-specific expression using transgenic soybean expressing GmMATE1*^C08^*pro-*GUS*. The GUS stain could be observed clearer in soybean pods around the main vein, compared to seeds and the seed coat (Figure 4). As an additional confirmation, we also expressed the same construct in transgenic *A. thaliana*. The GUS stain was found mainly in the funicle of siliques (the equivalents of soybean pods in *A. thaliana* [34]) (Figure 4). The funicle is the compartment through which the secondary metabolites are transported into developing seeds [35].

### 2.4. Manipulation of GmMATE1 in Soybean Significantly Altered the Isoflavone Contents in Seeds

After GmMATE1 had been shown to facilitate the transport of isoflavones in both the aglycone and glycoside forms in yeast, we further investigated its function in the native soybean system by knocking out the native GmMATE1 gene using the CRISPR/Cas9 system or by overexpressing GmMATE1 under the control of a constitutive 35S promoter.

**Figure 5:**
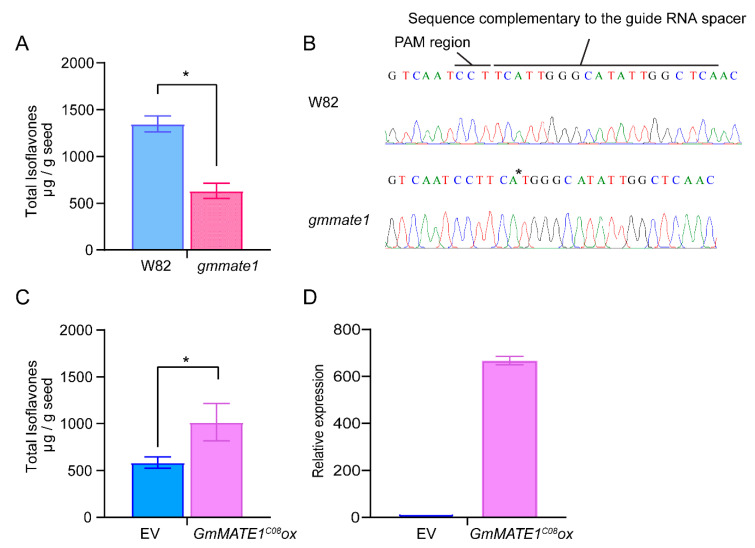
GmMATE1 is responsible for determining seed isoflavone contents. (**A**) The total isoflavone contents in the CRISPR/Cas9-edited GmMATE1 mutant was significantly lower than those in its wild-type, W82. Values shown were the means of at least three independently collected batches of seeds ± SEM. Significant differences were determined by Mann-Whitney test. *, *p* < 0.05. (**B**) The mutation site in the GmMATE1*^C08^* gene, with the single-nucleotide deletion marked with an asterisk. (**C**) The levels of isoflavones in the transgenic soybean overexpressing GmMATE1*^C08^* were significantly higher than those in the empty vector control, both in the W82 background. Values shown were the means of four independently collected batches of seeds ± SEM. Significant differences were determined by Mann-Whitney test. *, *p* < 0.05. (**D**) Relative expression levels of GmMATE1 in the GmMATE1*^C08^-*overexpressor and the empty vector control in the W82 background. The expression levels were normalized to the reference gene, *VPS*, using the 2^−^^ΔΔCt^ method.

A guide RNA, targeting the first exon of GmMATE1, was used to generate knockout mutants in the soybean germplasm, Williams 82 (W82). After screening, *mate1* knockout lines were generated in the W82 background (Figure 5). The allelic form of GmMATE1*^C08^* is the same as that of W82. After propagating to the next generation, we obtained a homozygous GmMATE1 mutant in W82, carrying a single-nucleotide deletion of the gene, causing frameshift and resulting in the truncation of the protein product (Figure 5). Significantly lower total isoflavone contents were detected in the knockout mutant compared to the wild type (Figure 5). 

To assess the potential enhancement of isoflavone accumulation by increasing GmMATE1 expression, GmMATE1*^C08^* was overexpressed in soybean germplasm W82. A statistically significant enhancement in total isoflavones (genistein, daidzein, glycitein, genistin, daidzin, glycitin, malonylgenistin, malonyldaidzin, and malonylglycitin) was observed (Figure 5), suggesting an overall improvement in isoflavone accumulation.

## 3. Discussion

Soybean seeds are rich in secondary metabolites and are unique in containing high contents of isoflavones. While soybean seeds with high isoflavones are preferred for their health benefits [36], in the food industry, soybean seeds with lower isoflavone contents are better received by consumers due to their more pleasant taste [37]. A better understanding of the key players regulating isoflavone accumulation in soybean seeds is therefore important for designing strategies to manipulate the isoflavone contents. One of the missing links in this research area is the transporters that facilitate the accumulation of isoflavones in seeds.

In a previous study, we have identified *MATE* transporter genes in a QTL, regulating the total phenolics, flavonoids, and anti-oxidation activities [30]. We selected two genes, GmMATE1 and GmMATE2, for further analyses since they are both expressed in soybean seeds and exhibit allelic differences between the wild soybean W05 and the cultivated soybean C08 [30].

Using the yeast uptake system, we demonstrated that GmMATE1 and GmMATE2 are isoflavone transporters. To our best knowledge, it is the first functional test of isoflavone transporter genes from soybean.

In the yeast uptake assay, the increased uptake of the aglycone form of isoflavones was clearly shown in yeast expressing either GmMATE1 or GmMATE2. When feeding the yeast with glycosides, both aglycones and glycosides were detected in the transgenic yeast cells (Figure 2). We speculate that the uptake of isoflavones in yeast by MATE transporters could be a two-step process: First, isoflavone glycosides were taken up by the yeast into the cytosol. The glycosides were then metabolized to aglycones. GmMATE transporters in transgenic yeast cells then compartmentalized the aglycones, and potentially also the glycosides, into the vacuole. However, we were unable to detect any malonylglycosides using the uptake assay (Figure S5). Since there was no baseline level of transport, the yeast system might not be suitable for investigating potential malonylglycosides transport by GmMATE transporters.

The yeast uptake results also showed that the non-synonymous mutations on different alleles of either GmMATE1 or GmMATE2 from the soybean accessions C08 or W05 did not cause obvious differences in the transporter activities (Figure 1 and Figure 2). Therefore, such allelic differences are not directly related to the differences in the total contents of phenolics and flavonoids as well as the levels of anti-oxidation activities between C08 and W05 [30]. 

We also investigated the spatial expression patterns of GmMATE1 and GmMATE2. Gene expressions of GmMATE1 and GmMATE2 were detected in developing pods, seeds, and the seed coat, and were diminished when the seed matured (Figure 4). GUS assays in transgenic soybean and transgenic *A. thaliana* confirmed the differential gene expression in pods and in the tissues connected to pods and seeds (Figure 4). Both GmMATE1 and GmMATE2 were localized in the vacuolar membrane (Figure 3). These data strongly suggested that GmMATE1 and GmMATE2 are transporters regulating isoflavone accumulation in soybean seeds, presumably in storage vacuoles [38].

We further performed loss-of-function and gain-of-function tests for GmMATE1 using CRISPR-mutated soybeans and overexpressing transgenic soybean lines. The overall isoflavone accumulation was reduced in a GmMATE1 frameshift mutant and increased in transgenic soybeans overexpressing GmMATE1 (Figure 5). 

The overexpressors exhibited a slight but still significant improvement in isoflavone accumulation compared to the empty vector control. It is possible that the ectopic constitutive expression of GmMATE1 had disturbed the sink-source relationship and thus limited the improvement in seed isoflavone accumulation. While this evidence supports that GmMATE1 is a *bona fide* isoflavone transporter, this system could not be used effectively to study substrate specificity, since soybean pods and seeds may contain metabolic enzymes that could transform isoflavones from one form to another.

## 4. Materials and Methods

### 4.1. Plant Materials and Growth Conditions

Soybean mutants, transgenic soybeans, and their respective wild types used in the same experiments were grown in a greenhouse setting. The pods, seeds, and seed coats of the soybean accessions C08 and W05 for gene expression studies were harvested from soybean plants grown in an experimental field. Transgenic *A. thaliana* in the Col-0 background expressing *GUS* was generated using the Agrobacterium-mediated transformation method [39]. The transgenic *A. thaliana* plants were grown in soil in a growth chamber (22 °C, 16 h/8 h light/dark cycle). The aerial part of the two-month-old transgenic *A. thaliana* plants were subjected to a GUS stain.

### 4.2. Gene Coning, Vector Construction and Transformation

The coding sequence (CDS) of GmMATE1*^C08^* (Glyma.19G120200) and GmMATE2*^C08^* (Glyma.19G120300) were cloned from mixed cDNAs of the pod and seed of C08, and GmMATE1*^W05^* (Glysoja.19G051265) and GmMATE2*^W05^* (Glysoja.19G051266) were cloned from mixed cDNAs of the pod and seed of W05. First-strand cDNAs were synthesized from RNAs with the SuperScript™ III First-Strand Synthesis System (18080051, ThermoFisher Scientific, Waltham, MA, USA) according to the manufacturer’s protocol. For the subcellular localization experiment using tobacco BrightYellow-2 (BY-2) cells, the target sequences were amplified from the cDNAs using Phusion High-Fidelity DNA polymerase (N0530S, New England Biolabs Inc, Ipswich, MA, USA). The full-length YFP cDNA was subcloned into pGWB2 as a control. The PCR products were first cloned into the pENTR/D-TOPO vector (K240020, ThermoFisher Scientific, Waltham, MA, USA) and then subcloned into pGWB41 [40] in frame with a C-terminal YFP through the LR reactions (11791020, ThermoFisher Scientific, Waltham, MA, USA). The confirmed plasmids were transformed into tobacco BY-2 cells using *Agrobacterium tumefaciens* LBA4404 [41]. 

For yeast uptake experiments, the pGBKT7 vector was first amplified with specific primers to remove the GAL4 DNA binding domain sequence, producing the pGBKT7∆BD plasmid. The GmMATE1*^C08/W05^* or GmMATE2*^C08/W05^* PCR product was cloned into pGBKT7∆BD between the *Xma*I and *Sal*I restriction sites and transformed into *E. coli* (DH5α) for plasmid amplification. pGBKT7∆BD-GmMATE plasmids were transformed into the Y2HGold (630489, Takara, Shiga, Japan) yeast strain using the LiAc/PEG method [42]. The transformants were selected on the synthetic dropout medium without tryptophan (SD-Trp). Yeast transformed with just pGBKT7∆BD was used as the empty vector (EV) control.

To generate expression constructs for soybean transformation, the GUS reporter downstream of the cauliflower mosaic virus (CMV) 35S promoter of the plasmid pCambia3301 was replaced with the CDS of GmMATE1*^C08^* in frame with a C-terminal 3x *FLAG* tag between the *Nco*I and *Bst*EII sites to generate the construct pCambia3301 *P35S::GmMATE1^C08^-3x FLAG* (*GmMATE1^C08^ox*). The pCambia3301 *P35S::3x FLAG* (EV) was used as the control. 

To generate the GUS reporter construct, the 35S promoter of the pCambia3301 plasmid was released using restriction enzymes *Bam*HI and *Nco*I and replaced with the sequence 2.5 kb upstream of the translational start site of GmMATE1*^C08^*. 

To generate genome-edited constructs, the guide RNA spacer specific to GmMATE1 was cloned into pBlu/gRNA (Addgene plasmid #59188) as previously described [43]. The pBlu/gRNA plasmid was provided by Robert Stupar [44]. After that, the guide RNA cassette in the pBlu/gRNA was released with *Xba*I and *Eco*RV and introduced into pFGC-pcoCas9 (Addgene plasmid #52256) between the *Xba*I and *Sma*I sites. The pFGC-pcoCas9 plasmid was provided by Dr. Jen Sheen (Harvard Medical School). Target fragments of GmMATE1 were amplified from the genomic DNA of each mutant line using the DreamTaq polymerase kit (EP0702, ThermoFisher Scientific, Waltham, MA, USA) to amplify the GmMATE1 gene variants, which were then cloned into the pMD20 T-vector (3270, Takara, Shiga, Japan) for TA cloning. A collection of 10 to 20 colonies were randomly picked for Sanger sequencing to detect the occurrence of mutation.

The constructs for generating transgenic soybeans or mutants were transformed into the soybean accession Williams 82 (W82) using a cotyledonary-node method with the *Agrobacterium tumefaciens* strain EHA105 [45].

All clones were confirmed by Sanger sequencing. Primers used for cloning are listed in Supplementary Table S1.

### 4.3. Yeast Uptake Assay

Yeast transformed with the expression vectors were grown in 50 mL SD-Trp at 30 °C with shaking at 220 rpm until OD600 of the cells reached 2.0. Yeast transformed with the empty pGBKT7∆BD was used as the negative control in the uptake assay. The uptake assay was performed as previously described [46] with these modifications. In brief, the cells were pelleted at 3000× *g* for 5 min, and then resuspended in 5 mL of 1× phosphate-buffered saline (PBS buffer, pH7.4). Each 1-mL aliquot of cells was then treated with a mixture of 200 μM flavonoids or isoflavones at 30 °C with shaking at 220 rpm overnight. The treated yeast cells were pelleted by centrifugation at 3000× *g* for 5 min and then washed twice with 50 mM potassium phosphate buffer (pH 7.8). The yeast cells were lysed using the freeze-thaw method, and intracellular metabolites were extracted with pure methanol as previously described [47]. Briefly, glass powder and 500 µL pure methanol at −80 °C (quenching process), together with 50 µM formononetin as spike, were added to the yeast cells. After vortexing for 5 s, the cells were frozen in liquid nitrogen and then thawed on ice. The freeze-thaw cycle was repeated once. After pelleting the cell debris, the extract was filtered through an Acrodisc 13 mm minispike 0.2 µm PVDF filter (4450, Pall Corporation, Port Washington, NY, USA). Five microliters of the extract were injected into a UPLC-PDA and separated through an Acquity BEH C18 reverse-phase column (2.1 mm × 50 mm, 1.7 µm) (186002350, Waters Corporation, Milford, MA, USA). The mobile phase for separation was a mixture of solvent A (0.5% formic acid [*v*/*v*] in water) and solvent B (0.5% formic acid [*v*/*v*] in acetonitrile). The flow rate was set at 0.5 mL/min. The gradient was set as 0–1 min, 5–10% solvent B; 1–7 min, 10–30% solvent B; 7–10 min, 30–45% solvent B; 10–11.5 min, 45–100% solvent B; 11.5–13.5 min, 100–5% solvent B. Retention of the metabolites were detected with UV absorbance at 260 nm [48]**,** and the areas under the curve were integrated using the software Empower2 Waters. The quantity of the target metabolite was calculated against the respective standard curve with R^2^ > 0.999 and corrected with the formononetin spike.

### 4.4. Extraction of Seed Isoflavones

Mature soybean seeds were weighed, and isoflavones were extracted as previously reported [49] with minor modifications. Briefly, 1 g of the sample was ground into powder in liquid nitrogen, and 10 mL of 80% (*v*/*v*) methanol was used for extraction. The mixture was vortexed and then extracted at 4 °C overnight. The debris was then removed by centrifugation at 8500 rpm at 4 °C for 15 min. Before the UPLC-PDA injection, the extract was filtered through an Acrodisc 13 mm minispike 0.2 µm PVDF filter (4450, Pall Corporation, Port Washington, NY, USA). UPLC-PDA analysis of the isoflavones was carried out as mentioned in Section 4.3. The identification and quantification of nine isoflavones were performed by comparing against known standards. Daidzin (021096), glycitin (GL-002), genistin (021050), malonylgenistin (06-1454), daidzein (D-101), glycitein (GL-001), and genistein (G-103) were purchased from Indofine Chemical Company, Inc., Hillsborough, NJ, USA. Malonyldaidzin (PS1633-0010) was purchased from Chengdu Push Bio-Technology Co., Ltd., Chengdu, Sichuan, China and malonylglycitin (139-13831) was purchased from Fujifilm Wako Pure Chemical Corporation, Chuo-ku, Osaka, Japan.

### 4.5. Subcellular Localization of GmMATE1 and GmMATE2 in Tobacco BrightYellow-2 (BY-2) Cells

The suspension cultures were stained with FM^TM^4-64 dye (ThermoFisher Scientific, Waltham, MA, USA Ex: 559 nm, Em: 575–675 nm) for 24 h for the visualization of the vacuolar membrane [50]. A collection of 10 to 20 cells were randomly picked from each of the three biological replicates for analysis. Images were captured immediately after staining before FM^TM^4-64 internalization and 24 h after staining for visualization of the plasma membrane and vacuolar membrane, respectively. The images were processed by Leica SP8 Confocal Microscopy.

### 4.6. Expression of GmMATE1 and GmMATE2 in Pod, Seed, and Seed Coat

Total RNA was extracted from the soybean pod, seed, and seed coat using Fruit-mate (9192, Takara, Shiga, Japan), supplemented with RNasin^®^ (recombinant, N2515, Promega, Madison, WI, USA), and TRIzol™ Reagent (15596018, ThermoFisher Scientific, Waltham, MA, USA) according to the manufacturers’ instructions. Tissues from three individual soybean plants were pooled as one biological replicate (including seed coat and seed). Two biological replicates were collected in total. For the transgenic soybean overexpressing GmMATE1*^C08^*, total RNA was extracted from the pod collected at 60 days after flowering (DAF). To remove DNA contamination, all RNA samples were treated with RNase-free DNase I (18068015, ThermoFisher Scientific, Waltham, MA, USA) according to the manufacturer’s protocol for the downstream experiment.

One-Step TB Green^®^ PrimeScript^TM^ RT-PCR Kit II (Perfect Real Time) (RR086A, Takara, Shiga, Japan) was used for RT-qPCR on a CFX96 Touch Real-Time PCR Detection System (BioRad, Hercules, CA, USA) according to manufacturers’ instructions. In brief, 15 ng of DNase-I treated RNA was used in each 20-µL reaction together with 0.4 µM of each primer, 2X One step buffer, and 0.8 µL of Prime-Script One step enzyme mix. The reaction protocol began with reverse transcription at 42 **°**C for 5 min, heat deactivation at 95 **°**C for 10 s, followed by 40 cycles of denaturation at 95 **°**C for 5 s and annealing and extension at 55 **°**C for 30 s. Three technical repeats were performed for each reaction. *VPS* was used as the reference gene for normalization [51]. The 2^−∆Ct^ and 2^−∆∆Ct^ methods were applied to calculate the relative expression levels for the endogenous genes and ectopically expressed gene, respectively. All primers used are listed in Supplementary Table S1.

### 4.7. β-Glucuronidase (GUS) Staining 

Transgenic soybean pod and seed and transgenic *A. thaliana* expressing the GUS reporter under the control of the GmMATE1*^C08^* promoter (−2.5 kb) were used for the GUS histochemical assay as previously described [52]. The GUS staining buffer contained 1 mM X-gluc (5-bromo-4-chloro-3-indolyl-β-D-glucuronide), 100 mM NaPO4 (pH7.0), 10 mM EDTA (pH 7.0), 0.5 mM ferricyanide, and 0.5 mM ferrocyanide. The tissues were vacuum infiltrated on ice with the GUS staining solution for 10 min. Next, the tissues were incubated in the GUS staining solution in the dark at 37 °C overnight. The samples were then removed by a series of incubation with absolute ethanol at room temperature until the complete removal of chlorophyll. Images of the stained tissues were visualized by camera. 

## 5. Conclusions

GmMATE1 and GmMATE2 are isoflavone transporters localized in the vacuolar membrane. They are potentially responsible for the transportation of genistein, daidzein, and glycitein in the soybean pod and seed. Allelic forms of GmMATE1 and GmMATE2 from the soybean germplasm W05 or C08 showed no significant differences in the various isoflavones accumulated under the same yeast assay system. GmMATE1 was differentially expressed in the developing seed pod. Removing GmMATE1 in soybeans resulted in lowered seed isoflavone contents while overexpressing the same gene-increased seed isoflavone contents. This supports the notion that GmMATE1, and possibly also GmMATE2, are *bona fide* isoflavone transporters.

## Data Availability

All data generated in this study are available within this manuscript and companion Supplementary Materials.

## Supplementary Materials

The following are available online at https://www.mdpi.com/article/10.3390/ijms222112017/s1.
