# Supplementary material for: MATE-Type Proteins Are Responsible for Isoflavone Transportation and Accumulation in Soybean Seeds
Source: Int J Mol Sci. 2021 Nov 6;22(21):12017. doi: 10.3390/ijms222112017 (PMC8585119; doi:10.3390/ijms222112017)
Supplement: Supplementary file 1 [file ijms-22-12017-s001.zip › ijms-1433268-supplementary.pdf]

Int. J. Mol. Sci.

MATE-type proteins are responsible for isoflavone transportation and accumulation in soybean seeds

Ming-Sin Ng[1], Yee-Shan Ku[1,*], Wai-Shing Yung[1], Sau-Shan Cheng[1], Chun-Kuen Man[1], Liu Yang [1], Shikui Song[2], Gyuhwa Chung[3], and Hon-Ming Lam[1,*]

1 Centre for Soybean Research of the State Key Laboratory of Agrobiotechnology and School of Life Sciences, the Chinese University of Hong Kong, Hong Kong SAR, PR China;
2 Institute of Advanced Agricultural Sciences, Peking University, Beijing 100871, PR China;
3 Department of Biotechnology, Chonnam National University, Yeosu 59626, Korea
* Correspondence: ysamyku@cuhk.edu.hk; honming@cuhk.edu.hk

Supplementary Materials

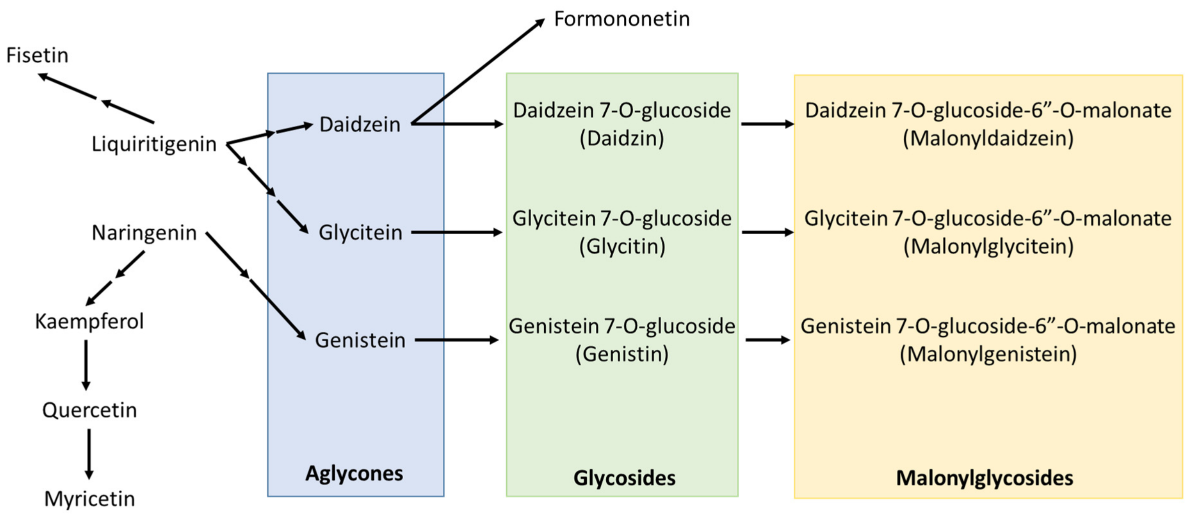

Figure S1. A schematic diagram showing the biosynthesis of the flavonoid-related compounds used in yeast uptake assays in this study [1].

1. Ferreyra, M. L. F.; Rius, S. P.; Casati, P. Flavonoids: biosynthesis, biological functions, and biotechnological applications. *Front. Plant Sci.* **2012,** *3*, 222.

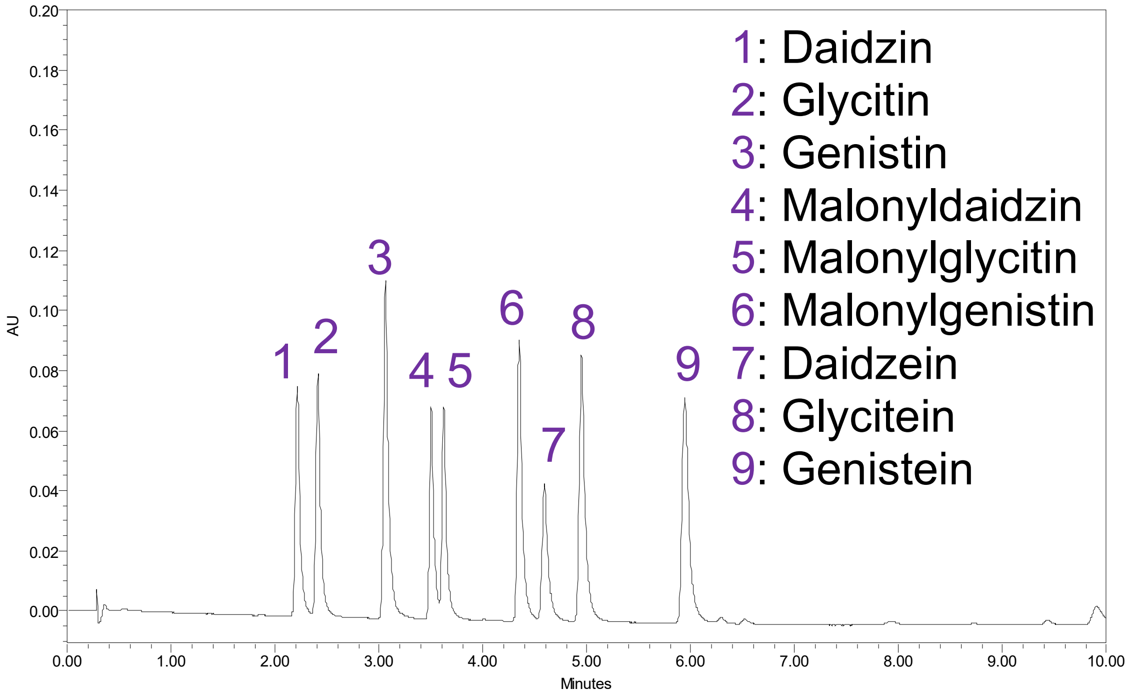

Figure S2. A UPLC chromatogram showing the absorption peaks of the nine isoflavone standards. 32ng of each chemical was injected into the UPLC system.

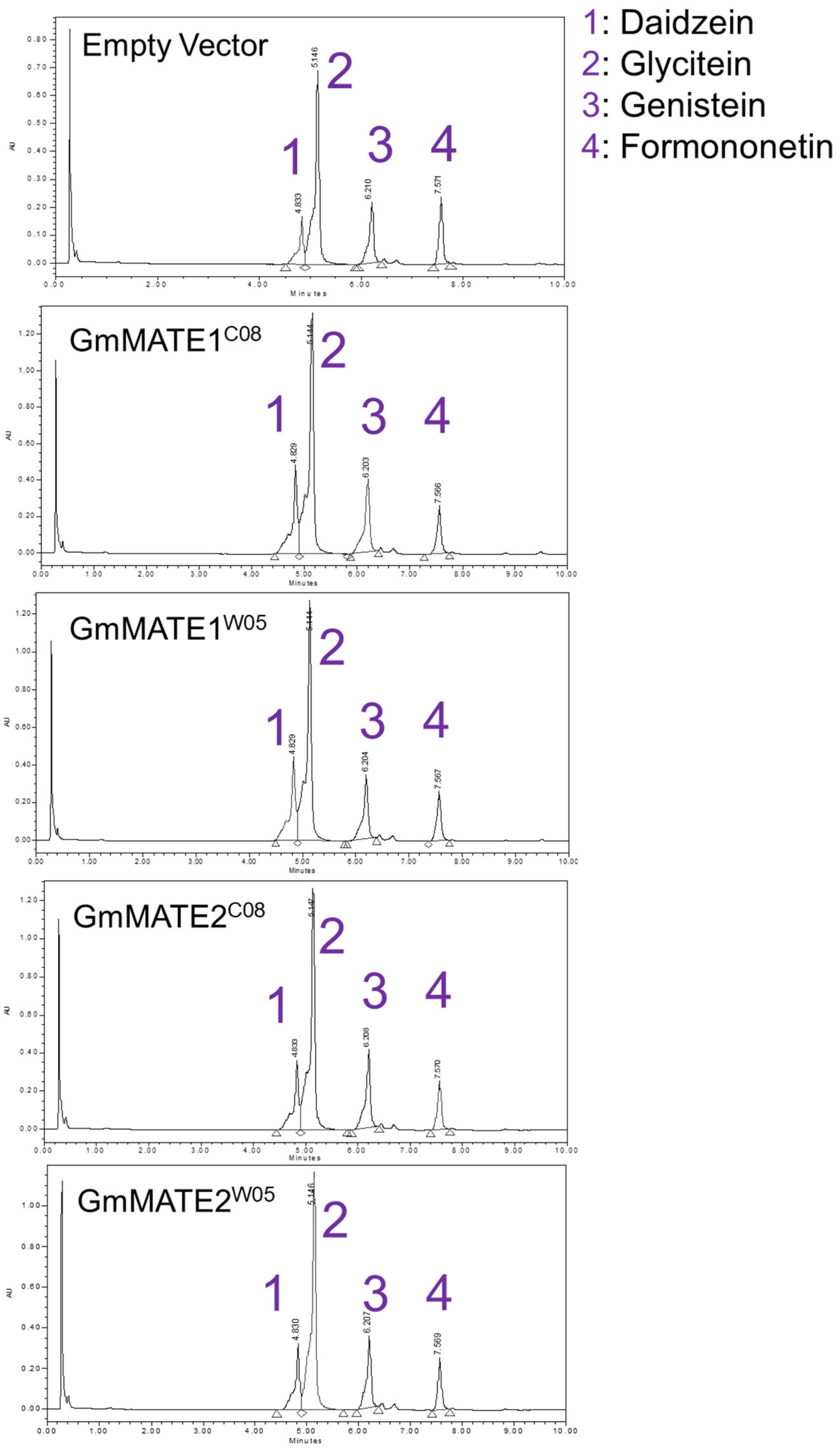

Figure S3. Representative UPLC chromatograms of the extracts from transgenic yeasts fed a mixture of isoflavone aglycones. The yeasts ectopically expressing *GmMATE1* or *GmMATE2* from cultivated soybean C08 or wild soybean W05, and yeasts transformed with the empty vector, were fed a mixture of 200μM each of daidzein, genistein, and glycitein. Pure methanol was used for the extraction of intracellular metabolites in yeast. A representative profile for each transgenic yeast is shown. Formononetin is used as the spike-in standard.

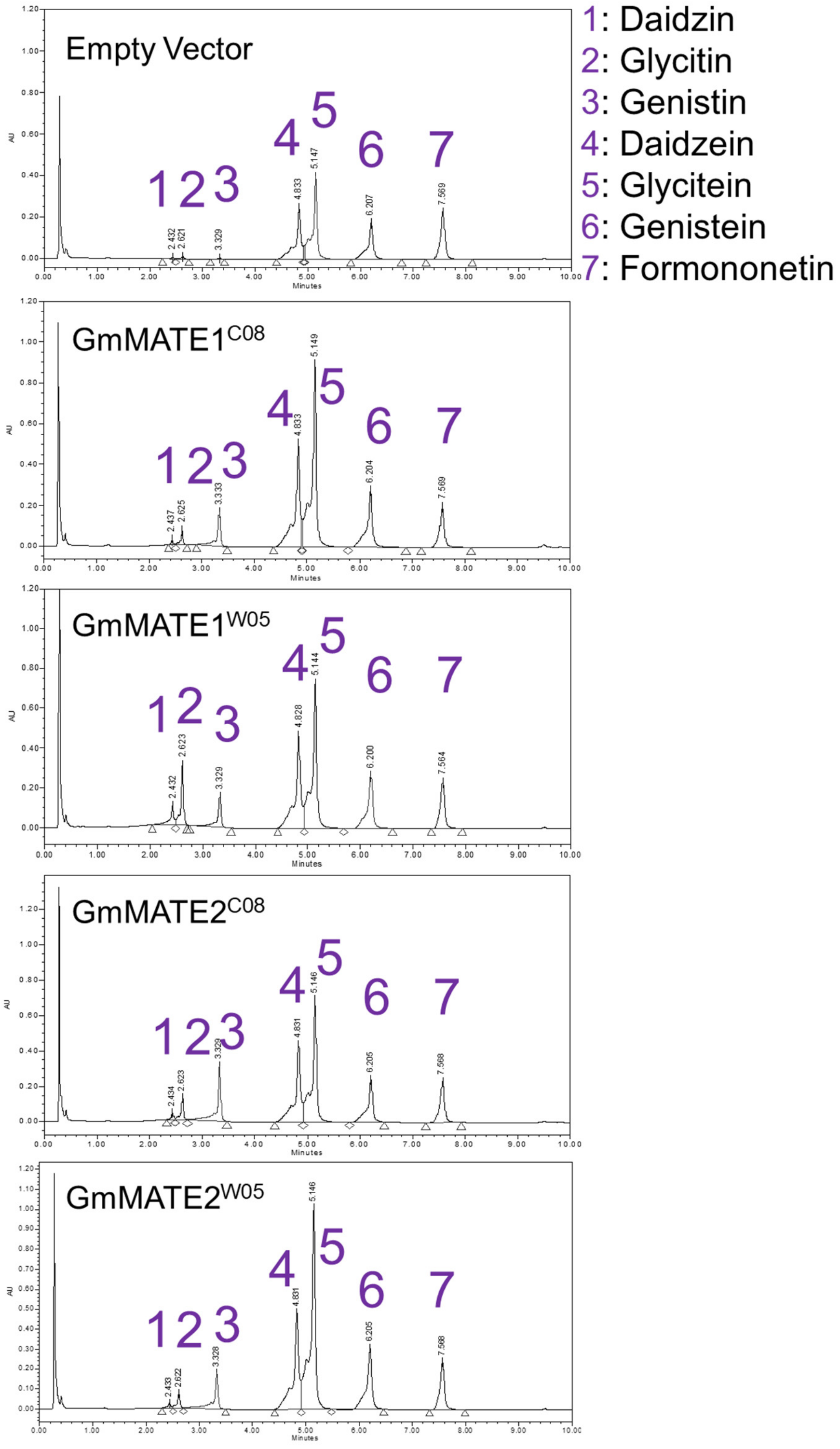

Figure S4. Representative UPLC chromatograms of the extracts from transgenic yeasts fed a mixture of isoflavone glycosides. The yeasts ectopically expressing *GmMATE1* or *GmMATE2* from cultivated soybean C08 or wild soybean W05, and yeasts transformed with the empty vector, were fed a mixture of 200μM each of daidzin, genistin, and glycitin. Pure methanol was used for the extraction of intracellular metabolites in yeast. A representative profile for each transgenic yeast is shown. Formononetin is used as the spike-in standard.

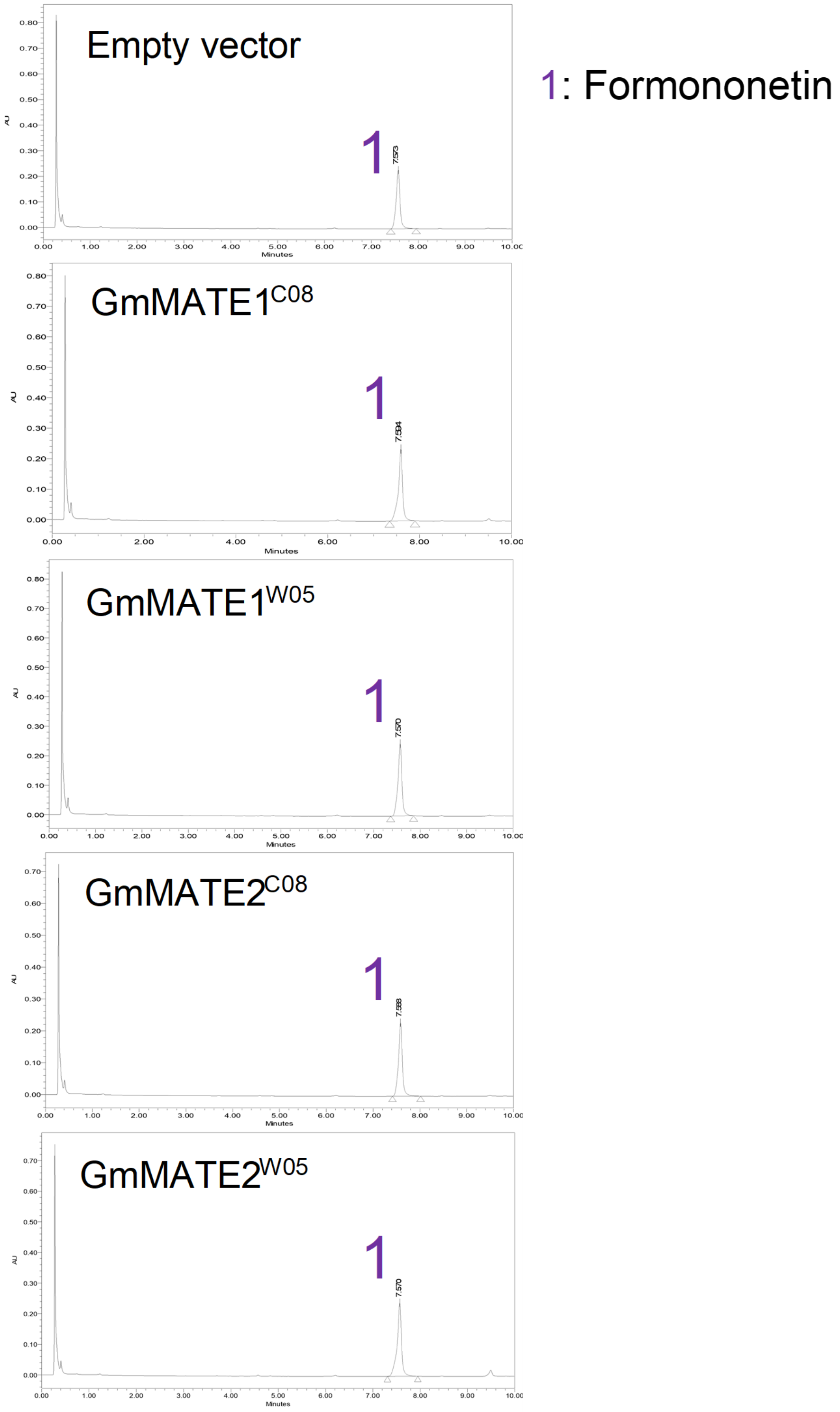

Figure S5. Representative UPLC chromatograms of the extracts from transgenic yeasts fed a mixture of malonylated isoflavones. The yeasts ectopically expressing *GmMATE1* or *GmMATE2* from cultivated soybean C08 or wild soybean W05, and yeasts transformed with the empty vector, were fed a mixture of 200μM each of malonylgenistin, malonyldaidzin and malonylglycitin. Pure methanol was used for the extraction of intracellular metabolites. Formononetin is used as the spike-in standard. Similar results were obtained from four independent experiments.

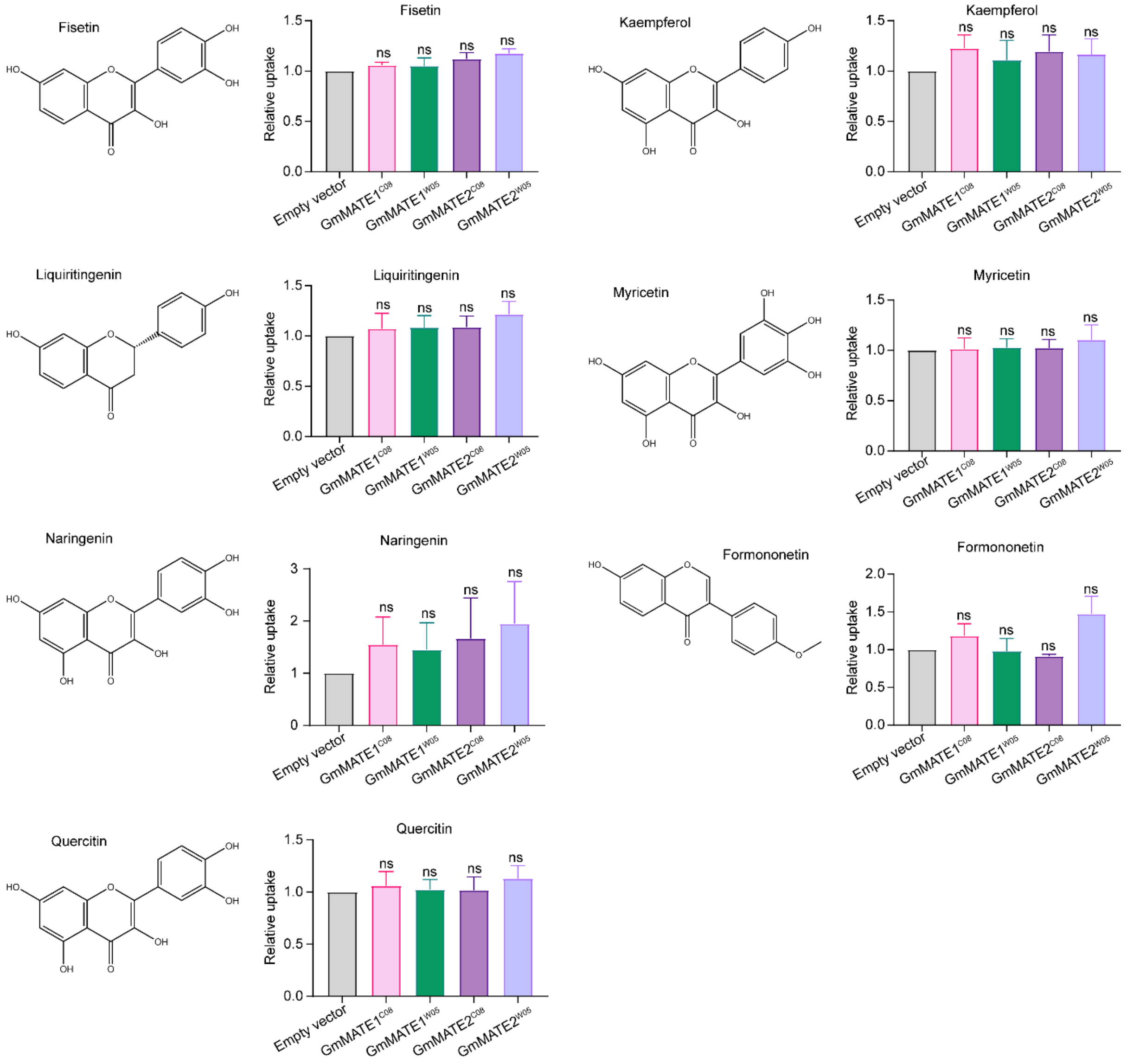

Figure S6. Yeast uptake assays with a mixture of flavonoid-related compounds. Metabolites were extracted from yeast after feeding for 24 hours and analysed using UPLC against the respective standards. Values obtained from the yeast expressing *GmMATE1* or *GmMATE2* were normalized to those from the empty vector control (EV) of the same experiment. Values shown were the means of three independent experiments ± SEM. Significant differences compared to EV were determined by Mann-Whitney test. ns, not significant.

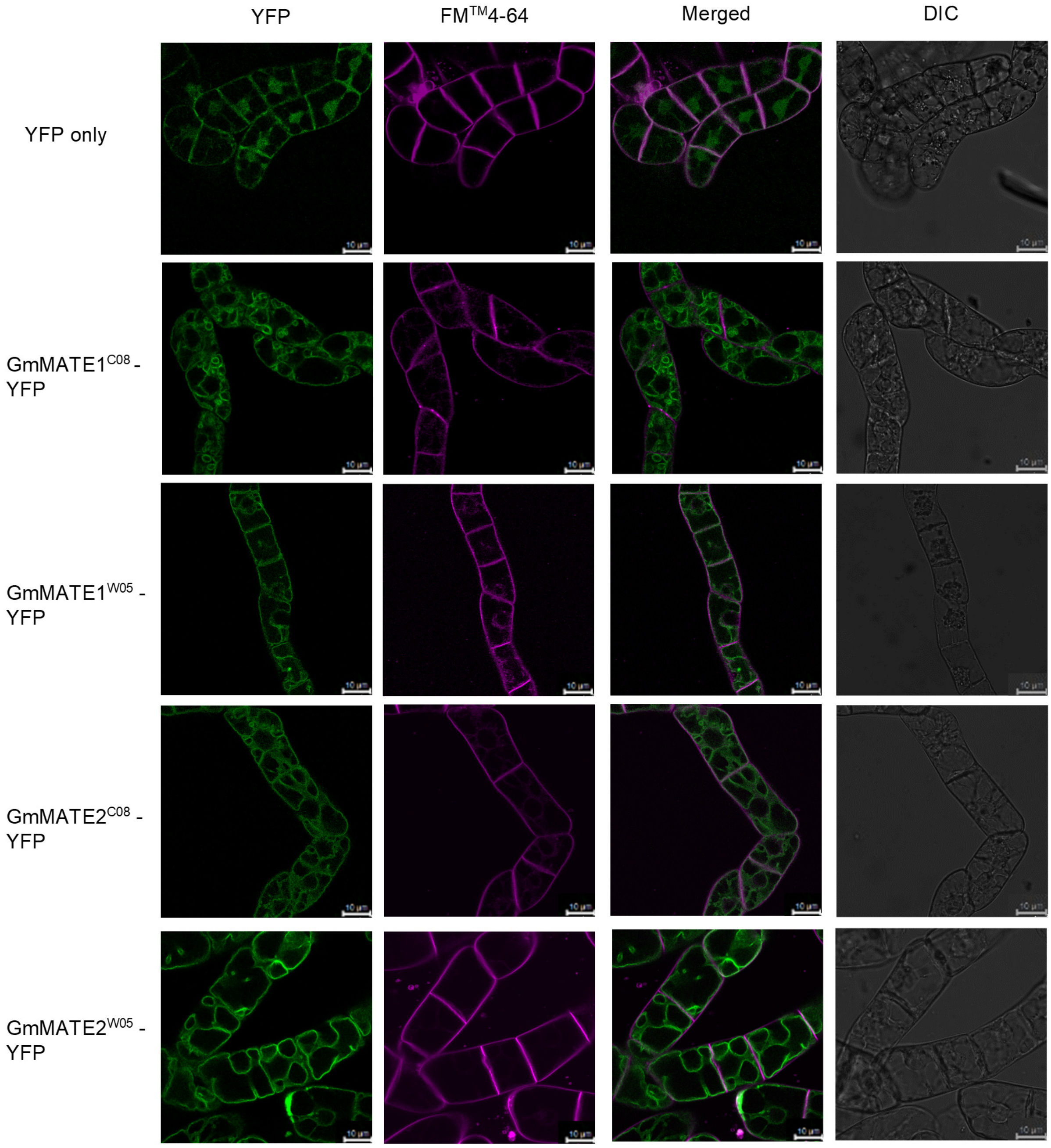

Figure S7. Subcellular localization of GmMATE1 and GmMATE2 in tobacco (*Nicotiana tabacum*) BrightYellow-2 (BY-2) cells. *GmMATE1* or *GmMATE2* was fused with *YFP* (Ex: 514 nm, Em: 525-547 nm) and expressed in BY-2 cells under the control of a cauliflower mosaic virus (CaMV) 35S promoter. The transgenic BY-2 cells were stained with FM^{TM}4-64 (Invitrogen, Ex: 559 nm, Em: 575-675 nm). Photos were taken immediately after staining and before the internalization of the stain. Photos were taken with an SP8 confocal microscope. Ten to 20 cells were randomly picked from each of the three biological replicates for analysis. Scale bar = 10 µm.

Table S1. List of primers used in this study.

Primer name	Primer sequence (5' to 3')
**For RT-qPCR**	
GmMATE1 Forward	TTTGTGGGCAAGCATATGGG
GmMATE1 Reverse	CCAGCAACTTGAGCAATGGT
GmMATE2 Forward	GAAACAAAGAGAGAAGAACATC
GmMATE2 Reverse	CTAGTGAATATGGCTGGTG
VPS Forward	AAAGAGTCTCATCCCACAAC
VPS Reverse	CGCATATTCCCAATCTCAGA
**For constructing yeast vectors**	
GmMATE1 cloned into pGBKT7ΔBD Forward (with *Xma*I cutting site)	ACCCGGGAATGGAAATGGAAGAGGA
GmMATE1 cloned into pGBKT7ΔBD Reverse (with *Sal*I cutting site)	AAAGTCGACCTAGCTAGATATGCTTGATCTACTG
MATE2 cloned into pGBKT7ΔBD Forward (with *Xma*I cutting site)	ACCCGGGAATGGAGGGGCATCTAAAG
MATE2 cloned into pGBKT7ΔBD Reverse (with *Sal*I cutting site)	AAAGTCGACTTAGCTAACATATTTTCTTTGTGC
pGBKT7 whole vector ΔBD Forward	Phos-CCGGAATTTGTAATACGACTCACTATAGGGC
pGBKT7 whole vector ΔBD Reverse	Phos-CTTTCAGGAGGCTTGCTTCAAGCTTGGAGTTG
**For CRISPR/Cas9 vector construction**	
CRISPR_pBlu gRNA for *GmMATE1* Forward	AAAGAAGACCTGTTTTGAGCCAATATGCCCAATGAGT TTTAGAGCTAGAAATAGCAAG
CRISPR_pBlu gRNA for *GmMATE1* Reverse	GGGCTGCAGGAATTCAAA
**For overexpression vector construction**	
$GmMATE1^{C08}$ Forward (with *Nco*I cutting site)	AAACCATGGATGGAAATGGAAGAGGAGC
$GmMATE1^{C08}$ Reverse (with *BstE*II cutting site)	AAAAAGGTCACCCTACTTGTCATCGTCATCCTTGTAGT CGATGTCATGATCTTTATAATCACCGTCATGGTCTTTG TAGTCGCTAGATATGCTTGATC
**For GUS reporter construct**	
$GmMATE1^{C08}$ native promoter Forward (with *BamH*I site)	AAAAGGATCCTTTTTTTAAAATTTATTAAAAAAAAAAGT TACTCAAC
$GmMATE1^{C08}$ native promoter Reverse (with *Nco*I site)	AAAAAGATCTACCATGGTGCTACTATAGAGCTGTCAAT GTCTTATTG
**For the construction of plasmids for subcellular localization study**	
GmMATE1 Forward	CACCATGGAAATGGAAGAGGAGCTAAAGG
GmMATE1 Reverse	GCTAGATATGCTTGATCTACTG
GmMATE2 Forward	CACCATGGAGGGGCATCTAAAGCAGAAGC
GmMATE2 Reverse	GCTAACATATTTTCTTTGTGCTTC
YFP Forward	CACCATGGTGAGCAAGGGCGAGGA
YFP Reverse	TTACTTGTACAGCTCGTCCATGCCGAG
